# Supplementary material for: Brain-state mediated modulation of inter-laminar dependencies in visual cortex
Source: Nat Commun. 2024 Jun 14;15:5105. doi: 10.1038/s41467-024-49144-w (PMC11178935; doi:10.1038/s41467-024-49144-w)
Supplement: Supplementary file 1 — Supplementary Information [file 41467_2024_49144_MOESM1_ESM.pdf]

Figure S1

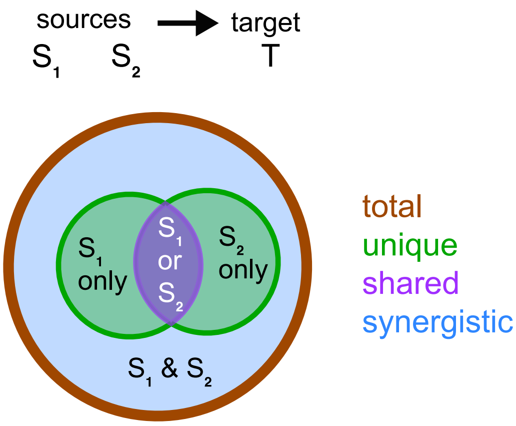

Figure S1 Information decomposition in a multi-variate system
Complete Partial information decomposition (PID) framework[35,36] based definition of types of information that multiple sources can have about a target.

Figure S2

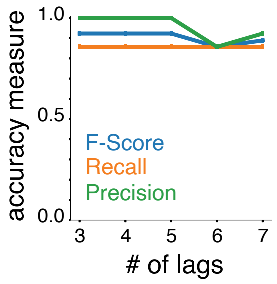

Figure S2 Additional MTwDBN performance metric for synthetic data
Recall, precision and F-score measures for weighted DAGs fit to synthetic data illustrated in Fig 2e as a function of number of lags.

Figure S3

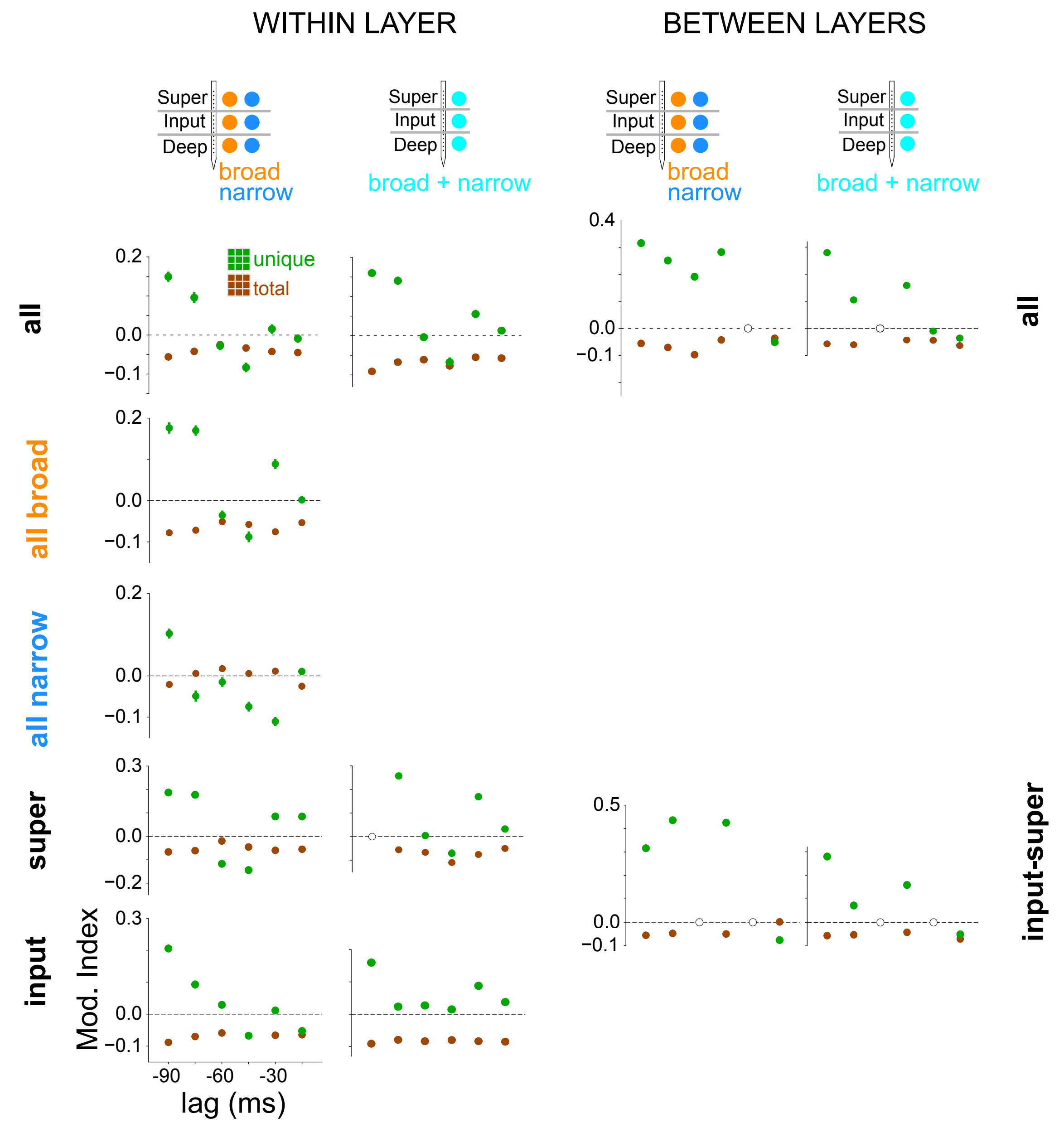

Figure S3 Modulation of dependencies in a V4 laminar network across attention conditions. MTwDBN-based modulation (green) of all unique dependencies between the laminar populations. Modulation of the same dependencies as estimated by logistic regression (brown). Open circles indicate no significant edges for a given time lag. Sche matic at the top of every column depicts the populations used for identifying laminar dependency structure within (left) and between (right) cortical layers. *broad*, *narrow*: cell-class specific populations identified by spike shape (see SI Methods).

Figure S4

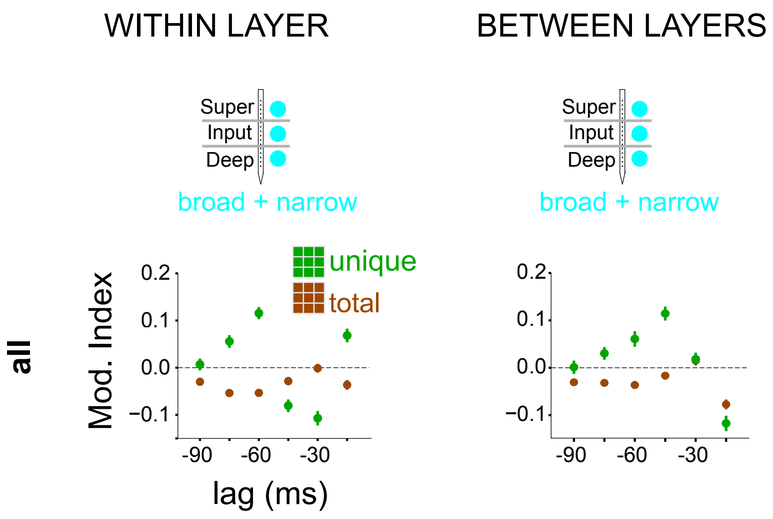

Figure S4 Modulation of dependencies in a V4 laminar network across behavioral outcomes at perceptual threshold. MTwDBN-based modulation (green) of all unique dependencies between the laminar populations. Modulation of the same dependencies as estimated by logistic regression (brown). Schematic at the top of every column depicts the populations used for identifying laminar dependency structure within (left) and between (right) cortical layers.

Figure S5

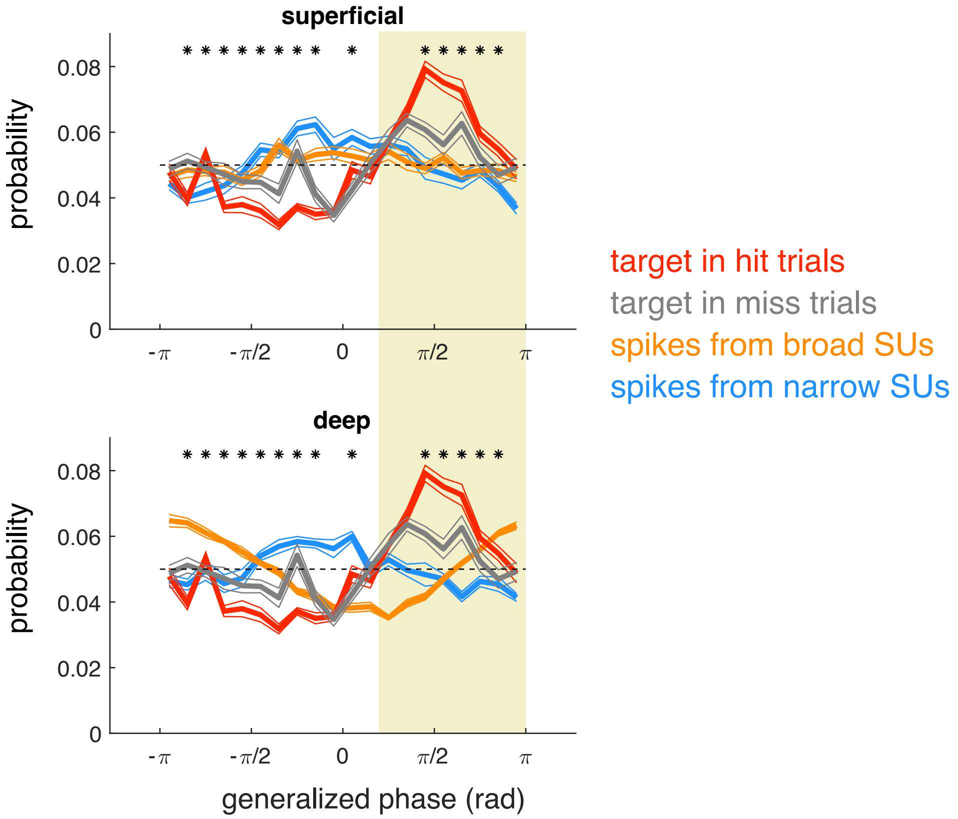

Figure S5 Spike probability in the superficial (top panel) and deep layer (bottom panel) as a function of generalized phase of the LFP, separately estimated for putative excitatory or broad spiking (orange), and putative inhibitory or narrow spiking (blue) SUs. Overlaid is target stimulus presentation probability as a function of generalized phase of the LFP (adjusted for cortical delay), separated by HIT (red) and MISS (grey) trials. Asterisk (*) indicates phases with significant difference between the two trial types (Ranked sum test, corrected for multiple comparison).
